# Bridging immune-neurovascular crosstalk via the immunomodulatory microspheres for promoting neural repair

**DOI:** 10.1016/j.bioactmat.2024.10.031

**Published:** 2024-11-08

**Authors:** Tongtong Xu, Lin Gan, Wei Chen, Dandan Zheng, Hanlai Li, Shiyu Deng, Dongliang Qian, Tingting Gu, Qianyuan Lian, Gracie Shen, Qingzhu An, Wanlu Li, Zhijun Zhang, Guo-Yuan Yang, Huitong Ruan, Wenguo Cui, Yaohui Tang

**Affiliations:** a Department of Orthopaedics and School of Biomedical Engineering, Shanghai Key Laboratory for Prevention and Treatment of Bone and Joint Diseases, Shanghai Institute of Traumatology and Orthopaedics, Ruijin Hospital, Shanghai Jiao Tong University School of Medicine, Shanghai Jiao Tong University, 197 Ruijin 2nd Road, Shanghai, 200025, China; b Department of Neurosurgery, Huashan Hospital, Shanghai Medical School, Fudan University, 12 Wulumuqi Middle Road, Shanghai, 200040, China; c Loomis Chaffee School, 4 Batchelder Road, Windsor, CT, 06095, USA

**Keywords:** Immune modulating microsphere, Ischemic stroke, Crosstalk, Angiogenesis, Neurogenesis

## Abstract

The crosstalk between immune cells and the neurovascular unit plays a pivotal role in neural regeneration following central nervous system (CNS) injury. Maintaining brain immune homeostasis is crucial for restoring neurovascular function. In this study, an interactive bridge was developed via an immunomodulatory hydrogel microsphere to link the interaction network between microglia and the neurovascular unit, thereby precisely regulating immune-neurovascular crosstalk and achieving neural function recovery. This immunomodulatory crosstalk microsphere (MP/RIL4) was composed of microglia-targeted RAP12 peptide-modified interleukin-4 (IL-4) nanoparticles and boronic ester-functionalized hydrogel using biotin-avidin reaction and air-microfluidic techniques. We confirmed that the immunomodulatory microspheres reduced the expression of pro-inflammatory factors including IL-1β, iNOS, and CD86, while upregulating levels of anti-inflammatory factors such as IL-10, Arg-1, and CD206 in microglia. In addition, injection of the MP/RIL4 significantly mitigated brain atrophy volume in a mouse model of ischemic stroke, promoted neurobehavioral recovery, and enhanced the crosstalk between immune cells and the neurovascular unit, thus increasing angiogenesis and neurogenesis of stroke mice. In summary, the immunomodulatory microspheres, capable of orchestrating the interaction between immune cells and neurovascular unit, hold considerable therapeutic potential for ischemic stroke and other CNS diseases.

## Highlights

• Immunomodulatory microspheres (MP/RIL4) held great anti-inflammatory effects *in vitro* and *in vivo*.
• MP/RIL4 mitigated brain atrophy volume and promoted neurobehavioral recovery for a long-time of ischemic stroke mice.
• MP/RIL4 increased angiogenesis and neurogenesis via enhancing immune-neurovascular crosstalk of ischemic stroke mice.

## Graphical abstract

The immune-neurovascular crosstalk bridge via the immunomodulatory microsphere (MP/RIL4) was constructed, which could induce the transformation of microglia into an anti-inflammatory phenotype, and link their interaction with neurovascular unit, ultimately succeeding to promote angiogenesis and neurogenesis in ischemic stroke model mice.

**Figure:**
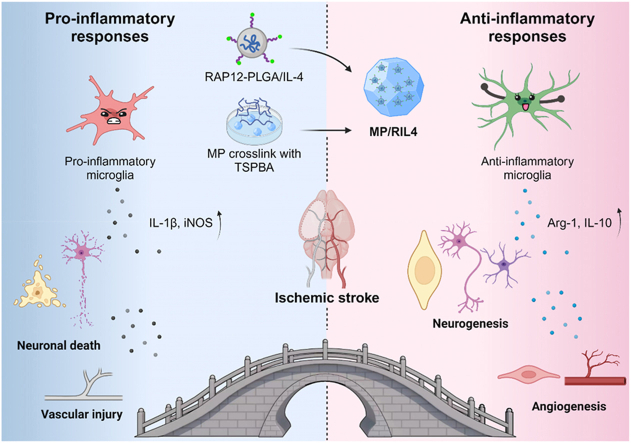

## 1 Introduction

Various cell types and tissues communicate through intricate signaling networks, often referred to as “crosstalk”, which is vital for maintaining normal tissue growth, development, and repair following injuries [1]. For instance, under normal circumstances, muscles and tendons collaborate to handle mechanical loads [2]. When the muscle-tendon junction or skeletal muscle is injured, effective signal communication among muscle cells, fibroblasts, immune cells, and blood vessels is imperative for promoting further repair and regeneration [3,4]. In addition, immune system closely interacts with neurovascular unit to maintain neural homeostasis [[5], [6], [7]]. Immune cells such as microglia prune synapses to sustain neural function and secrete angiogenic factors to promote blood vessel growth and vascular network formation [[8], [9], [10], [11]]. Disrupting the balance of crosstalk can lead to inflammatory responses, abnormal angiogenesis, loss of regeneration capacity, and ultimately hinder tissue remodeling [12,13]. Thus, establishing an interactive bridge between injured tissues through tissue engineering approaches is an urgent issue to be addressed [14,15].

When central nervous system (CNS) is injured, imbalance in the interaction between brain immune cells and neurovascular units is a key factor affecting brain regeneration [16,17]. The disruption of crosstalk between immune system and neurovascular unit in injured area causes the instability of immune microenvironment homeostasis, triggering inflammatory factor storm, leading to blood-brain barrier disruption, glial scar formation, increased oxidative stress, accumulation of neurotoxic substances, and dysfunction of the neurovascular unit, all of which inhibit neural regeneration [18,19]. Hence, signal transduction and interactions between immune cells and components of the neurovascular unit, including neurons, glial cells, and endothelial cells, are critical for modulating inflammatory responses and tissue repair. Microglia, the primary immune effector cells in the CNS, are polarized to anti-inflammatory type after brain injury, and secretes a range of neurotrophic elements such as transforming growth factor-β (TGF-β), brain-derived neurotrophic factor (BDNF) for promoting neural regeneration, and vascular endothelial growth factor (VEGF) to contact with endothelial cells to stimulate angiogenesis, enhancing cerebral blood flow, thus facilitating neural function recovery [[20], [21], [22]]. Therefore, promoting microglial polarization towards an anti-inflammatory phenotype after CNS injury is beneficial for strengthening the immune-neurovascular interaction network to promote neural repair. Interleukin-4 (IL-4) is a classic therapeutic factor and plays a critical role in promoting microglial transition to an anti-inflammatory phenotype [23]. However, systemic treatment based on IL-4 suffers from limitations due to its short half-life and inefficient delivery, requiring multiple and high-dose administration [24,25]. To address these limitations, Chen et al. developed an “immune-responsive hydrogel” that can release therapeutic factors in response to local MMP2 and MMP9 levels, adjusting microglial activation states and reducing inflammation, yet it lacks precisely targeted modulating microglia phenotype and fails to effectively link immune cells with the neurovascular unit [26]. Our previous work developed a nano-biosensor by synthesizing a RAP12 peptide that could targeted regulate microglia, and reduce neuroinflammation [27]. However, without structural support and intervention in the local abnormal microenvironment, it is challenging to sustain microglia in their long-term anti-inflammatory polarized condition [28]. Thus, there is an urgent need to develop biomaterials that can not only induce microglial polarization in a long-term targeted manner, but also orchestrate microglia with the neurovascular system to facilitate immune-neurovascular crosstalk and promote neural function recovery.

Stroke is the predominant cause of mortality and morbidity [29,30]. Regulating crosstalk between brain immunity and neurovascular unit is crucial for the enhancement of neural function recovery after stroke. Herein, an interactive bridge was developed via an immunomodulatory hydrogel microsphere to connect the interaction network between microglia and the neurovascular unit, thereby precisely modulating immune-neurovascular crosstalk and facilitating the restoration of neural function. The immunomodulatory microsphere (MP/RIL4) consisting of microglia-targeted RAP12 peptide-modified IL-4 nanoparticles and boronic ester-functionalized hydrogel was engineered by using biotin-avidin reaction and air-microfluidic techniques. In addition, this system could regulate the immune niches in a long-term manner through sustained release of IL-4 nanoparticles in response to the microenvironment, prolong the action time and therapeutic efficiency of IL-4 in the brain injury area, thereby increasing angiogenesis and neurogenesis, and ultimately promoting neurobehavioral recovery in ischemic stroke mice. We successfully fabricated the ROS-responsive and IL4-laden immunomodulator for targeted regulation of microglia, and verified its biocompatibility. We demonstrated that the immunomodulator increased the transition of microglia into an anti-inflammatory type, and reduced infarct volume, improved neurobehavioral recovery as well as neurovascular remodeling of stroke mice. Our findings provide a new strategy for developing biomaterials that restore the balance of tissue crosstalk.

## 2 Materials and methods

### 2.1 Animals

Animal experiments followed guidelines of the Institutional Animal Care and Use Committee (IACUC) of Shanghai Jiao Tong University, Shanghai, China. Male ICR mice (8-week-old, 24–26 g weight) of Specific-pathogen-free (SPF) grade were provided by Lingchang Biotechnology Co., Ltd., Shanghai, China. Mice were housed in the animal facility with 12-h light/12-h dark at 22 °C, 60 % humidity. Water and food were provided on time.

### 2.2 Construction and characterization of IL-4 loaded RAP12-PLGA nanoparticles

First, avidin-palmitate was constructed by the following procedure: 5 mg of avidin and 0.37 mg of palmitic acid-N-hydroxysuccinimide ester were dissolved and reacted together for 12 h at 37 °C in phosphate-buffered saline (PBS, Meilunbio, Dalian, China) containing 2 % sodium deoxycholate. 12 h later, the resulting product was purified by using the dialysis bag (*M*_*W*_ 8000–12,000) in 0.15 % sodium deoxycholate. Then, avidin-modified PLGA nanoparticles (PLGA-NPs) was prepared as previously described [31]. PLGA (50 mg/ml) was dissolved in 500 μl dichloromethane to form an oil solution, and then 200 μl of 10 μg interleukin-4 (IL-4) protein solution (Novoprotein, Shanghai, China) was dropped into the PLGA organic solvent solution and sonicated using SCIENTZ-IID (Scientz, Ningbo, China, 70 W, 1 min) to form the water-in-oil emulsion mixture. Then this mixture was incorporated into the 0.5 % polyvinyl alcohol solution (PVA) containing avidin-palmitate and sonicated to produce a double emulsion (70 W, 3 min). This avidin-PLGA solution was then put into 12 ml 0.5 % PVA solution, stirred for 4h to get rid of excessive organic solvent to form the uniform avidin-PLGA-NPs. The avidin-PLGA-NPs were collected through centrifugation (12,000 rpm, 4 °C, 25 min) and washed with double distilled water (ddH_2_O) to remove unencapsulated IL-4. The resulting product was resuspended with PBS and reacted with 1 mg biotin-modified RAP12 peptide (Apeptide Co., Ltd, Shanghai, China) for 1 h. Additional RAP12-biotin was separated through repeated centrifugation and resuspended in PBS containing 0.5 % trehalose to obtain the IL4-encapsulated RAP12-modified PLGA-NPs (RIL4), and was lyophilized and preserved at −20 °C for future use.

After construction, the size distribution was tested using dynamic light scattering (DLS, Malvern Instruments Ltd., UK). Briefly, 50 μl of the PLGA-NPs loaded with IL-4 (IL4-NP) before reacting with RAP12-biotin and 50 μl of the RIL4 were put into DLS respectively for size distribution. The morphology of IL4-NP and RIL4 was detected by scanning electron microscopy (SEM).

### 2.3 Preparation and characterization of PVA microspheres

Air-microfluidic technique was used to prepare PVA microspheres as previously reported [32]. First, dissolve a certain amount of PVA in dimethyl sulfoxide (DMSO) to obtain the 7.5 % PVA DMSO solution. Then utilizing the air-shear device to use the external N_2_ to shear the internal PVA solution into discrete droplets, which were then collected in anhydrous ethanol. Under the solvent exchange between DMSO and ethanol, the PVA droplets could be quickly dehydrated and form PVA hydrogel precursors via crosslinking. Then, the precursor microspheres were added into N^1^-(4-boronobenzyl)-N^3^-(4-boronophenyl)-N^1^, N^1^, N^3^, N^3^-tetramethylpropane-1, 3-diaminium (TSPBA) linker to form PVA microspheres (MPs) due to the Borate ester bond, and was lyophilized for further application [33]. The MP/RIL4 were prepared by mixing the RIL4 NPs with lyophilized MPs. The morphologies of PVA microsphere precursors, MPs and MP/RIL4 were observed by the optical microscope and SEM.

### 2.4 BV2 microglia culture and stimulation

BV2 microglia cell line (National Collection of Authenticated Cell Cultures, Shanghai, China) was seeded in Dulbecco's modified Eagle medium (DMEM, Meilunbio) with heat-inactivated Fetal Bovine Serum (FBS, 10 %, BioVision, Shanghai, China) and 1 % antibiotic (Meilunbio). 50,000 BV2 microglia were seeded in a 24-well plate for 12 h, and cocultured with MPs, RIL4, or MP/RIL4 for 1 h. Subsequently, the cells were treated with LPS (200 ng/ml, Sigma-Aldrich, St. Louis, MO) or PBS for 24 h to assess whether pretreatment with RIL4 influenced LPS-caused neuroinflammation *in vitro*. Neuroinflammation was assessed using quantitative real-time polymerase chain reaction (RT-PCR), immunostaining, and Western blot (WB).

### 2.5 Identification of cell proliferation and survival

BV2 microglia were seeded at density of 10,000 in a 24-well plate for 12 h, and cocultured with PBS, MP, RIL4, or MP/RIL4 for 1 h. 1-hour later, cells were treated with 200 ng/ml LPS, or PBS for 24 h, 48 h or 72 h. The cell viability was assessed using CCK-8 assay as previously described (Meilunbio). Cell survival was investigated by live and dead cell double staining using PI (in red) and calcein-AM (in green) with 2 ml PBS add 1 μl PI and 1 μl calcein-AM. The live and dead of cells were detected under microscope after 20 min.

### 2.6 Transient middle cerebral artery occlusion (tMCAO) surgery and stereotaxic injection of MP/RIL4

Surgery of tMCAO was conducted as previously reported [34]. Briefly, adult mice were anesthetized with 4–5% isoflurane and kept with 1.5–2% isoflurane in oxygen (RWD Life Science Co., Ltd., Shenzhen, China). The carotid arteries were carefully separated and ligatured, and then a 6-0 suture (Covidien, USA) coated with silicone was threaded through the external carotid artery and then retrogradely navigated into the internal carotid artery to reach the middle cerebral artery. Reperfusion was accomplished by removing the suture and loosening the ligation after a 90-min interval. We used laser Doppler system (Moor Instruments, Devon, UK) to test the blood flow of the mice. Successful surgery was confirmed when blood flow dropped less than 20 % of the initial level, and recovered over 80 %. The identical surgical technique was performed on sham mice without insertion and occlusion of the suture. Animals were sacrificed 7 days after tMCAO for immunostaining, and 14 days after tMCAO for immunostaining, RT-PCR and WB analysis. Sample size of each group was Sham (n = 10), IS (n = 10), IS-MP (n = 11), IS-RIL4 (n = 12) and IS-MP/RIL4 (n = 10) respectively, excluding dead mice (Supp. Table 1).

10 μl 5 % hyaluronic acid containing 400 ng IL-4 or MP/RIL4 were stereotaxically injected into the mouse with the following coordinates: anterior posterior (AP), 0.02 mm, media lateral (ML), 2 mm, and dorsal ventral (DV), −3 mm at 24 h post-tMCAO.

### 2.7 Immunostaining

Cells were collected and fixed with 4 % paraformaldehyde (PFA, Macklin, Shanghai, China) for 10 min before immunostaining. Brain tissues were put in 4 % PFA at 4 °C for 4–6 h, and changed to 30 % sucrose for 2 d to be fully dehydrated, and then rapidly frozen in isopentane at −80 °C for later use. Brain tissues were cut into 20 μm in thickness in microscope slides. Before immunostaining, slides were taken out of −80 °C and dried under a fan. After fixation in 4 % PFA for 10 min, the slices were then rinsed in PBS for 5 min, 3 times. Antigen retrieval was performed using pH 9.0 EDTA (Servicebio, Wuhan, China). Brain slices were cycled with an immunohistochemical pen or cells were incubated with 0.3 % Triton X-100 (Sigma-Aldrich) for 10 min, and 2 % bovine serum albumin (BSA, Meilunbio) at room temperature (RT) for blocking, then incubated with primary antibodies for 14–16 h at 4 °C, including IBA1 (1:200, Novusbio, Centennial, CO), iNOS (1:200, ABclonal, Wuhan, China), Arg1 (1:200, Abclonal), IBA1 (1:200, WAKO, Osaka, Japan), CD206 (1:100, Abcam, Cambridge, UK), CD86 (1:100, Thermo Fisher, Waltham, MA), CD11b (1:100, Biolegend, San Diego, CA), Ki67 (1:200, Abcam), CD31 (1:200, R&D, Emeryville, CA), GFAP (1:200, Abcam) and DCX (1:200, Abcam). 14–16 h later, slides were rinsed in PBS for 10 min, 3 times, and incubated with Alexa Fluor 488 or Alexa Fluor 594-conjugated secondary antibodies (1:400, Invitrogen, Waltham, MA) for 1 h at RT. Cells or brain slices were sealed with mounting medium containing DAPI (Beyotime, Jiangsu, China). Confocal microscopy (Leica, Wetzlar, Germany) was employed to acquire fluorescence images. For each mouse, imaging was conducted on three slices with a distance of 200 μm, and three fields per slice were used for calculation.

### 2.8 Quantitative real-time polymerase chain reaction (RT-PCR)

TRIzol (Invitrogen) was added into cells for RNA extraction. Mice were sacrificed at 14 days after tMCAO. Brain tissues were quickly placed in an ice-cold mouse brain matrix. Brains were subsequently cut into 2 mm coronal sections before and after Circle of Willis, took injured tissues, and placed in microtubes containing TRIzol to extract RNA. Total RNA was extracted from lysed cells or targeted brain regions. We used spectrophotometer (Nanodrop 1000, Thermo Fisher) to detect the concentration of RNA, and then reverse transcribed into cDNA using Synthesis SuperMix Kit (Yeason, Shanghai, China). RT-PCR was conducted utilizing SYBR Green Master Mix Kit (Yeason). Comparative CT method (2^−ΔΔCT^) was carried out to do semi-quantification analysis. mRNA levels were normalized to GAPDH of control group and the results were presented as fold change. The primer sequences were as table below:

**Table:** 

	Forward primer (5′-3′)	Reverse primer (5′-3′)
iNOS	CAGCTGGGCTGTACAAACCTT	CATTGGAAGTGAAGCGTTTCG
IL-1β	TCCAGGATGAGGACATGAGCAC	GAACGTCACACACCAGCAGGTTA
Agr-1	GAACACGGCAGTGGCTTTAAC	TGCTTAGCTCTGTCTGCTTTGC
IL-10	GGTTGCCAAGCCTTATCGGA	ACCTGCTCCACTGCCTTGCT
GAPDH	GGCAAATTCAACGGCACAGTCAAG	TCGCTCCTGGAAGATGGTGATGG

### 2.9 Western blot (WB) analysis

Cells were collected using cell scraper in protein lysis buffer. Bicinchoninic acid (BCA) protein assay kit (Meilunbio) was used to measure protein concentration. 40 μg proteins were loaded on 10 % (W/V) SDS-PAGE and electrophoresed. The gels were then transferred to PVDF membrane (Millipore) and cultivated with primary antibodies including Arg1 (1:500, Abclonal) and β-actin (1:1000, Invitrogen) at 4 °C in a shaker for over 16 h. The protein-loaded PVDF membrane was rinsed in TBST (Epizyme, Shanghai, China) 3 times, 10 min each time, and cultivated with HRP-conjugated anti-rabbit and anti-mouse IgG (1:4000, Invitrogen) for 1 h at RT, and reacted with enhanced chemiluminescent substrate (Meilunbio). Semi-quantification was performed utilizing ImageJ software (NIH, Bethesda, MD).

### 2.10 NO production assay

Total Nitric Oxide Assay kit (Yeason) was used to measure the nitrite released into microglial culture supernatants indirectly. 60 μL supernatants condensed in 3 kD ultrafiltration tubes (Millipore) were mixed with nitrate reductase and Griess reagent, incubated for 10 min at room temperature. Then, the absorbance was measured at 540 nm using a microplate reader. Results were analyzed according to standard curve.

### 2.11 Enzyme-linked immunosorbent assay (ELISA)

IL-10 expression was measured using ELISA kit (ABclonal) followed by the manufacturer's instruction. Briefly, standards and samples were put into 96-well ELISA plate coated with IL-10 antibodies, and then added biotinylated antibody and streptavidin-HRP orderly. After incubation and washing, TMB substrate was added to show the color depth, acid was added to stop the reaction and the absorbance value was measured. The concentration of sample was calculated according to the standard curve.

### 2.12 Cresyl violet staining

Brain atrophy was assessed using cresyl violet staining as previously reported [35,36]. The areas of both contralateral and ipsilateral hemispheres were measured using ImageJ. The calculation of brain atrophy extent (ΔS) was the discrepancy in area between affected (ipsilateral) hemisphere and unaffected counterpart (contralateral hemisphere). The interval distance separating a pair of consecutive brain sections was defined as the height (H). Brain atrophy volume (V) was calculated as follow: V = ∑H/3 × [ΔSn + (ΔSn × ΔSn+1)1/2 + ΔSn+1], where the reduced areas of two successive slices were denoted as ΔSn and ΔSn+1, respectively.

### 2.13 Neurobehavioral tests

Neurobehavioral tests were performed at 1, 3, 7, and 14 d post-tMCAO by a researcher who was not aware of the experimental design or any treatments [37].

**Modified neurologic severity score (mNSS).** The mNSS includes assessment of motor, sensory and balance function, which was graded on a scale from 0 to 14. 0 denotes normal status, whereas a higher score signifies a more severe injury. **Elevated body swing test (EBST).** EBST was conducted to assess motor asymmetry in the mice. The mice were lifted by their tails and observed for their turning behavior. A total of 20 repetitions were performed for each mouse. The results were recorded as the number of deflections to the right/total number of repetitions. **Hanging wire test.** It was performed to evaluate the mice's coordination abilities and upper limb strength. Mice were situated at the midpoint of a suspended wire, and the time was measured. Initially, each mouse was given a baseline score of 10. A score of 1 point was deducted for every fall from the wire. The test continued until the score reached 0 or the mouse spent 180 s on the wire.

### 2.14 Isolation and culture of primary neural stem cells (NSCs)

Primary NSCs were isolated from mice cortex on embryonic day 16. Briefly, the cortex was extracted from embryo and digested into single-cell suspension, and then cultured in medium of DMEM/F12 (Meilunbio) supplemented with 20 ng/ml of bFGF (PeproTech), 20 ng/ml of EGF (PeproTech), 1 % GlutaMAX (Gibco), 1 % B27 (Gibco), and 1 % antibiotic. Neurospheres were passaged using accutase (Gibco) every 3–4 days, and cells within the first 3 passages were utilized for experiment.

### 2.15 Tube formation assay and neural differentiation

BV2 microglia were seeded at a density of 8000 per well in 0.4 μm transwell inserts (Absin) for 12 h, and incubated with MP/RIL4 for 1 h. Cells were then treated with or without LPS, and transferred to wells containing human umbilical vein endothelial cells (HUVECs) or NSCs for 24 h. For tube formation assay, Matrigel (Yeason) was thawed at 4 °C overnight, and added to wells of 24-well plate. After 1 h, HUVECs were seeded onto the Matrigel-coated wells and covered with transwell inserts containing the BV2 microglia for 24 h. Tube formation was quantified using the Angiogenesis Analysis plugin of ImageJ (https://imagej.net/ij/plugins/index.html).

For neural differentiation test, single NSCs were seeded in Poly-D-lysine (PDL, Sigma-Aldrich)-coated 24-well plate, and changed with differentiation medium consisted of neurobasal medium (Gibco) containing 2 % B27, 1 % glutaMAX, and 1 % antibiotic. Cells were then covered with transwell inserts containing the BV2 microglia for 24 h. NSCs differentiation was observed at day 7.

### 2.16 Statistical analysis

All statistical evaluations were conducted using Prism 9.0 (GraphPad). Data were presented as the mean ± standard error of the mean (SEM). For comparative analyses, unpaired *t*-test were employed for two-group comparisons, one-way or two-way ANOVA, followed by Dunnett or Tukey multiple comparisons, were utilized for multiple groups. Statistical significance was determined by *P* < 0.05 (two-tailed).

## 3 Results and discussion

### 3.1 Construction and characterization of the immunomodulatory microspheres (MP/RIL4)

**Fig. 1:**
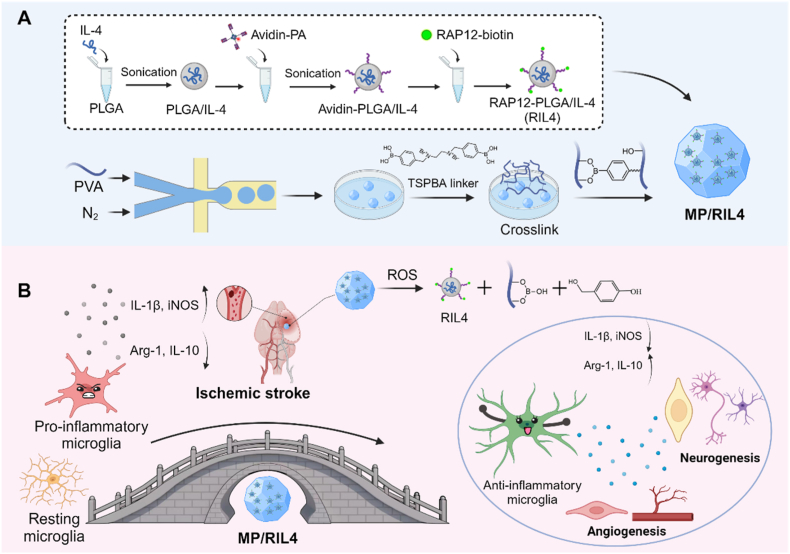
**Schematic diagram of immunomodulatory microsphere (MP/RIL4) for bridging immune neurovascular crosstalk.** (A) Preparation of the immunomodulatory microsphere (MP/RIL4). RAP12 modified nanoparticle with IL-4 was prepared via biotin-avidin reaction, and the boronic ester-functionalized PVA microspheres were fabricated using air-microfluidic technique and crosslinking with TSPBA. MP/RIL4 was obtained by adding the RIL4 nanoparticles into the lyophilized MP microspheres through negative pressure suction. (B) MP/RIL4 promoted the polarization of microglia to anti-inflammatory phenotype *in vitro* and *in vivo*, and improved neurogenesis and angiogenesis of ischemic stroke mice. The schematic diagram was created with BioRender.com.

**Fig. 2:**
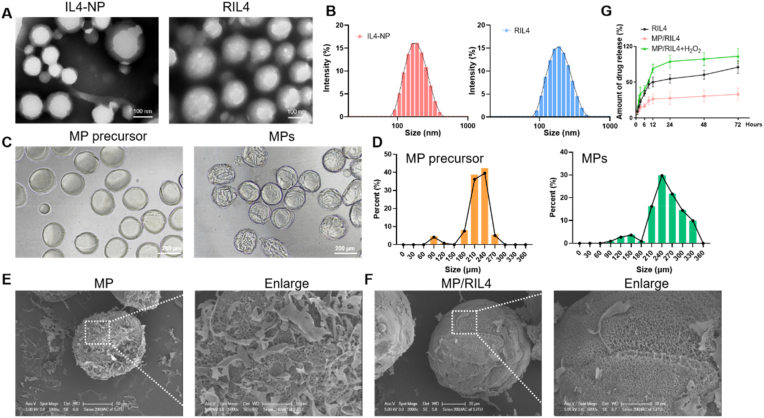
**Preparation and characterization of PVA microspheres loaded with RAP12-PLGA/IL-4 nanoparticles.** (A) Representative TEM images of nanoparticles loaded with IL-4 (IL4-NP), and IL4-NP modified with RAP12 (RIL4). (B) Quantitative size distribution analysis of IL4-NP and RIL4. (C, D) Bright filed images and quantitative size distribution analysis of PVA microsphere (MP) precursor and PVA microspheres (MPs). (E, F) Representative SEM images of MP and MP/RIL4. (G) Drug release of RIL4 in PBS, MP/RIL4 in PBS, and H_2_O_2_. n = 3. All data are presented as mean ± SEM.

Manufacturing and characterization of the immunomodulator are shown in Fig. 1. The preparation of RAP12-PLGA/IL-4 nanoparticles was prepared as our previous study described [27]. First, a double emulsion process was used to encapsulate IL-4, the immunomodulatory cytokine, into avidin-modified PLGA nanoparticles. Subsequently, the biotinylated RAP12 polypeptide was conjugated to the surface of avidin-modified nanoparticles via biotin-avidin interaction. TEM and DLS data indicated that both types of nanoparticles exhibited uniform spherical shapes, with a hydrodynamic diameter of approximately 180 nm. The size of free PLGA nanoparticle loaded with IL-4 was about 183 nm, and after modification with RAP12-biotin, the average size was 186 nm (Fig. 2A and B), indicating that the modification of RAP12 on the surface of IL4-NP did not affect its particle size. RIL4 NP held limitations of quick clearance, short half-life and inefficient delivery. To address these limitations, RIL4 NP was encapsulated in ROS-responsive PVA microspheres to enhance the retention of RIL4 NP and achieve the responsively prolonged release of IL-4. PVA-based microsphere precursor (MP precursor) was first produced by air-microfluid technique and solvent exchange of DMSO to ethanol. Then the MP precursor was crosslinked by adding the TSPBA linker to form the ROS-responsive PVA microspheres (MPs) through boronic ester reaction. Bright-field images showed the size distribution of MP precursor and MPs were both about 240 μm (Fig. 2C and D). Finally, the MP/RIL4 was obtained by adding the RIL4 nanoparticles into the lyophilized MP microspheres through negative pressure suction. SEM revealed that the MP/RIL4 presented a uniform spherical structure and a rougher surface than MP (Fig. 2E and F). RIL4 encapsulated with PVA microspheres in PBS exhibited a sustained release profile, and reached 40 % at 72 h, but had a rapid release in H_2_O_2_ solution (Fig. 2G). The results suggested that the immunomodulatory microspheres MP/RIL4 were ROS responsive and could achieve the sustained release of IL-4 in PLGA nanoparticles.

### 3.2 Biocompatibility identification of the “immunomodulator”

**Fig. 3:**
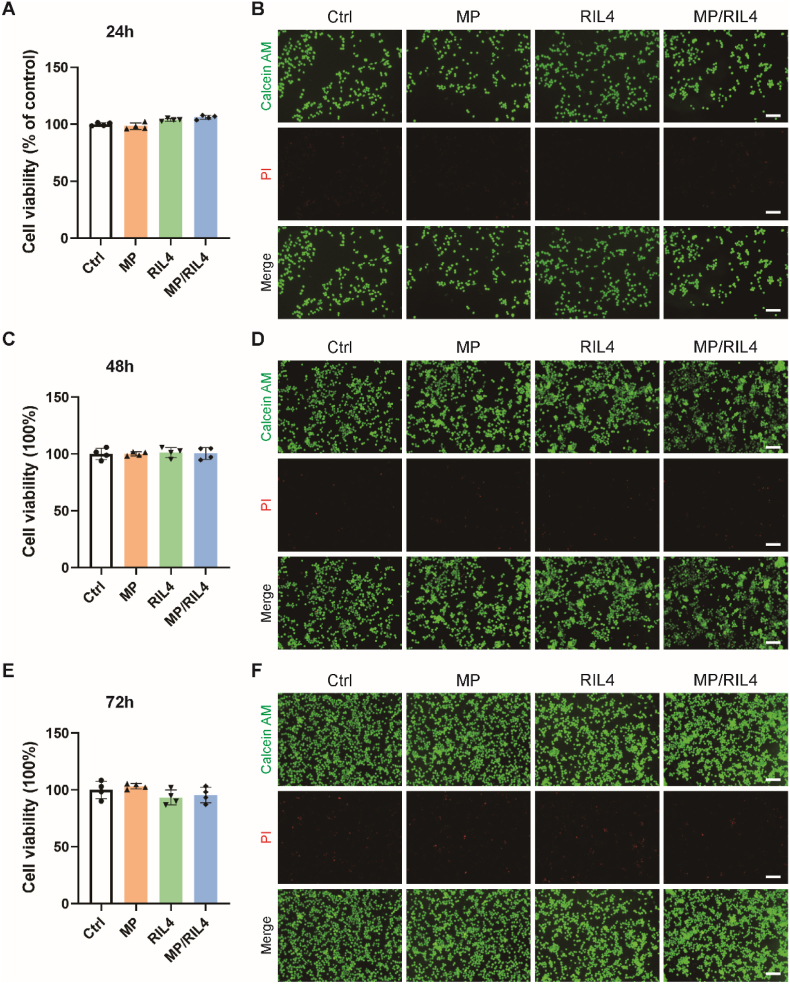
**Microspheres loaded with RAP12-PLGA/IL-4 did not affect the proliferation and survival of microglia.** (A) Cell viability of PBS, MP, RIL4 and MP/RIL4 co-cultured with BV2 microglia after 24 h n = 4. (B) Representative images of Calcein AM/PI staining images of PBS, MP, RIL4 and MP/RIL4 co-cultured with BV2 microglia after 24 h. Scale bar = 100 μm. (C) Cell viability of PBS, MP, RIL4 and MP/RIL4 co-cultured with BV2 microglia after 48 h n = 4. (D) Representative Calcein AM/PI staining images of PBS, MP, RIL4 and MP/RIL4 co-cultured with BV2 microglia after 48 h. Scale bar = 100 μm. (E) Cell viability of PBS, MP, RIL4 and MP/RIL4 co-cultured with BV2 microglia after 72 h n = 4. (F) Representative Calcein AM/PI staining images of PBS, MP, RIL4 and MP/RIL4 co-cultured with BV2 microglia after 72 h. Scale bar = 100 μm. All data are presented as mean ± SEM.

Biomaterials have been widely applied in treating CNS diseases and have demonstrated promising therapeutic outcomes [37,38]. However, biomaterials can potentially elicit a series of biological responses once contacting with living organisms, such as hematological reactions (platelet activation and thrombus formation), immune responses (activation of macrophages and lymphocytes, as well as adaptive immune responses), and tissue reactions (inflammatory response, fibrosis, and neovascularization) [[39], [40], [41]]. Consequently, when considering the clinical application of biological materials for therapeutic purposes, safety should be first addressed. To identify the biocompatibility of the “immunomodulator”, we used CCK8 to detect the cell viability and the calcein-AM/PI staining to determine the cell survival/death, wherein dead cells are PI (red) positive and live cells are calcein-AM (green) positive. We found that treating BV2 microglia with MP, RIL4 or MP/RIL4 for 24 h (Fig. 3A and B) or 48 h (Fig. 3C and D) did not affect the viability, even after 72 h in culture (Fig. 3E and F). These data demonstrated the great biocompatibility of the immunomodulator.

### 3.3 Immunoregulation effect of the “immunomodulator” *in vitro*

**Fig. 4:**
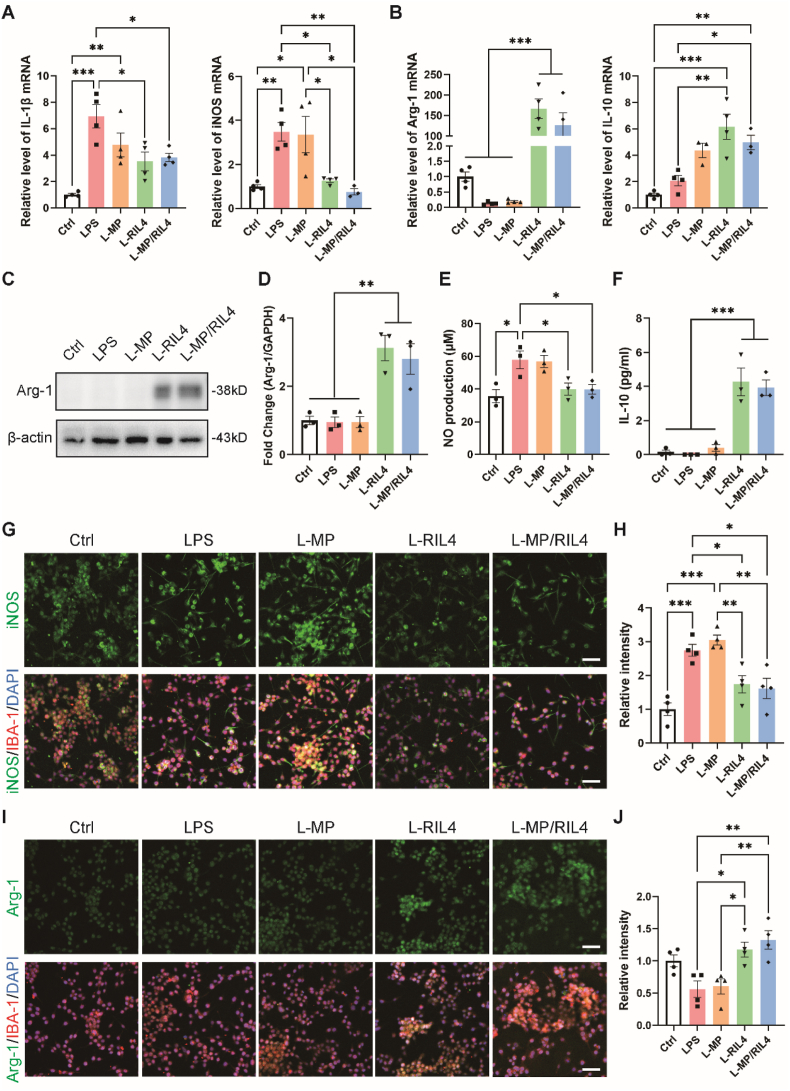
**Immunoregulation effect of the “immunomodulator” *in vitro*.** (A, B) Quantitative RT-PCR results of pro-inflammatory marker (IL-1β, iNOS) and anti-inflammatory marker (Arg-1, IL-10) expression of PBS, MP, RIL4 and MP/RIL4 co-cultured with LPS stimulated BV2 microglia after 24 h n = 3–4. (C, D) Western blot and quantitative analysis of Arg-1 expression after PBS, MP, RIL4 and MP/RIL4 co-cultured with LPS stimulated BV2 microglia after 24h. n = 3. (E) NO production of PBS, LPS, L-MP, L-RIL4 and L-MP/RIL4 co-cultured with BV2 microglia after 24 h measured by NO assay kit. n = 3. (F) IL-10 protein concentration of PBS, LPS, L-MP, L-RIL4 and L-MP/RIL4 co-cultured with BV2 microglia after 24 h determined by ELISA. n = 3. (G–J) Representative immunostaining images and relative total fluorescence intensity of iNOS, and Agr-1 with IBA-1 after PBS, MP, RIL4 and MP/RIL4 co-cultured with LPS stimulated BV2 microglia. Scale bar = 100 μm. n = 4. All data are presented as mean ± SEM, ∗*p* < 0.05, ∗∗*p* < 0.01, ∗∗∗*p* < 0.001.

Microglia exhibit a broad spectrum of states, from pro-inflammatory to anti-inflammatory [42,43]. It is well documented that IL-4 induces the polarization of microglia to anti-inflammatory phenotype, which has protective effects in numerous CNS diseases [23,44,45]. First of all, we detected the anti-inflammatory effect of the “immunomodulator” compared with naked nanoparticles and microspheres in normal cell culture conditions, and we found the up-regulation of Arg-1 and IL-10 in RIL4 and MP/RIL4 groups (Supp. Figs. 1A–B). We used lipopolysaccharide (LPS)-stimulated BV2 microglia to simulate neuroinflammation *in vitro*. We chose pretreating cells with drugs before LPS-stimulation, since it is reported that many drugs need time to reach an effective concentration within cells and to produce a biological effect [46,47]. BV2 microglia were co-cultured with MP, RIL4, and MP/RIL4 for 1 h and then stimulated with LPS (200 ng/ml) or PBS to detect if RIL4 pretreatment affected LPS-caused neuroinflammation. 24 h later, RT-PCR analysis revealed a substantial rise in pro-inflammatory markers including IL-1β and iNOS in groups with LPS compared to PBS group. RIL4 pretreatment reduced the expression of IL-1β and iNOS, and promoted the levels of anti-inflammatory indicators IL-10 and Arg-1 (Fig. 4A and B). Then, we employed WB to ascertain the anti-inflammatory impact of RIL-4 on BV2 cells at protein level. RIL4 treatment showed a remarkable increase in anti-inflammatory marker Arg-1 compared to LPS group (Fig. 4C and D). Since inducible nitric oxide synthase (iNOS) is largely expressed under LPS-stimulation and produces excess nitric oxide (NO), which aggravates cell death and inflammatory responses [48]. We used NO assay kit to measure the pro-inflammatory level. Results showed that L-RIL4 and L-MP/RIL4 groups decreased the level of NO in LPS and L-MP groups (Fig. 4E). We also performed the ELISA experiment to test the expression of anti-inflammatory cytokine IL-10, and we found that L-RIL4 and L-MP/RIL4 groups significantly increased the protein level of IL-10 (Fig. 4F). BV2 cells, which were pretreated with RIL4, manifested an outstanding anti-inflammatory effect. Additionally, immunofluorescence analysis showed that RIL4 pretreatment decreased iNOS and increased Arg-1 immunoreactivity (Fig. 4G–J).

RAP12 peptide was designed utilizing the high expression of lipoprotein receptor-related protein-1 (LRP1) on microglia [49,50]. Hence, this RAP12-modified nanoparticle (RIL4) is inclined to target and accumulate around microglia, which could enhance the polarization of microglia into anti-inflammatory phenotype. RIL4 nanoparticles may be internalized by microglia after binding, and they will not affect the function of loaded cargos as a commonly used and proven protein loading system [[51], [52], [53]]. Furthermore, IL-4 delivered into cells can also exert its effects through intracellular receptors or signaling molecules. To demonstrate the importance of RAP12, we added a control of MP/IL4 without RAP12 modification using immunostaining. We found that MP/IL4 without RAP12 could also reverse the pro-inflammatory level after LPS stimulation, but not as good as RIL4 or MP/RIL4 group (Supp. Figs. 2A–D). Overall, these results exhibited a superior anti-inflammatory effect of RIL4 *in vitro*.

### 3.4 Injection of the “immunomodulator” promoted neurobehavioral function and reduced brain atrophy volume of ischemic stroke mice

**Fig. 5:**
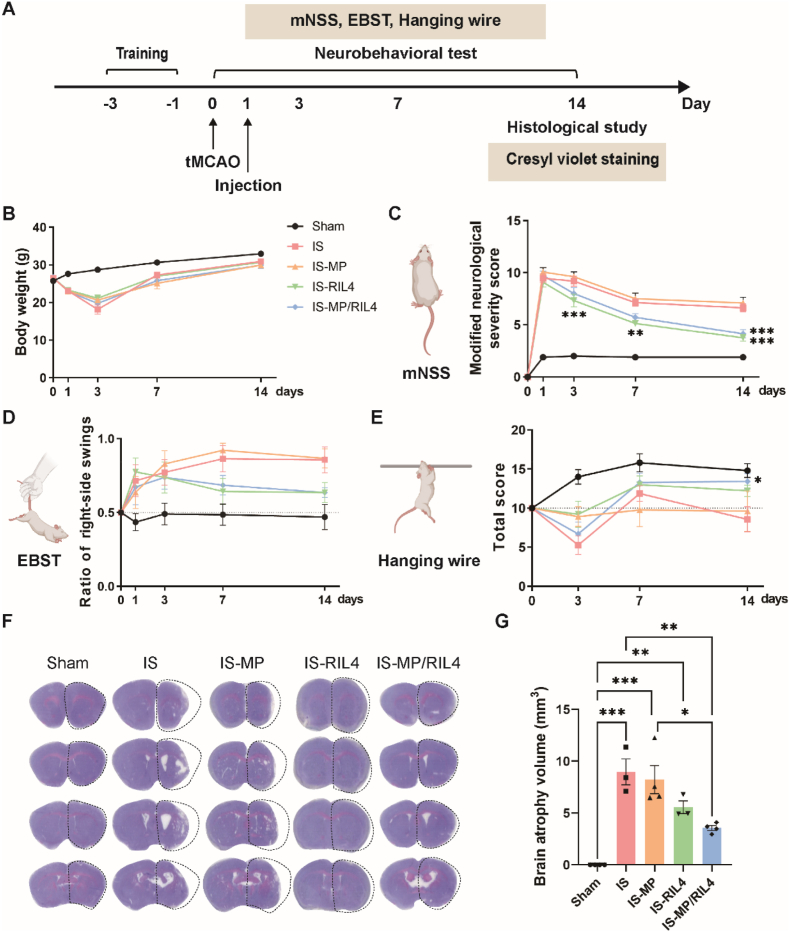
**The “immunomodulator” promoted neurobehavioral function and reduced brain infarct volume of ischemic stroke mice.** (A) Experimental diagram. (B) Analysis of body weight of each group. (C) mNSS analysis, (D) EBST, and (E) hanging wire test of ischemic mice. n = 7–15 mice. These mouse patterns were created with BioRender.com. (F, G) Brain atrophy volume was quantified by cresyl violet staining at 14 d after ischemic stroke. n = 3 mice. All data are presented as mean ± SEM, ∗*p* < 0.05, ∗∗*p* < 0.01, ∗∗∗*p* < 0.001.

Ischemic stroke often results in irreversible brain injury and subsequent brain atrophy [54]. Brain atrophy post-stroke arises from neuronal death in the core infarct area and secondary injuries caused by insufficient blood flow, involving processes such as small vessel occlusion, inflammatory responses, oxidative stress, and excitotoxicity [55]. Brain atrophy leads to structural reorganization and disruption of neuronal networks, significantly affecting neurobehavioral functions [56]. Neurobehavioral assessments are crucial in monitoring disease progression, and predicting prognosis [57]. We designed the study shown in Fig. 5A to detect the neurorestorative effect of our designed “immunomodulator”. Mice were allocated into five groups at random: (1) Sham group (non-treated with any surgery or drugs); (2) IS group (exposed to tMCAO surgery); (3) IS-MP group (Stereotaxic injection of PVA microspheres at 1 d after tMCAO); (4) IS-RIL4 group (Stereotaxic injection of RAP12-PLGA/IL-4 at 1 d after tMCAO); and (5) IS-MP/RIL4 group (Stereotaxic injection of PVA microspheres combined with RAP12-PLGA/IL-4 at 1 d after tMCAO). The mNSS, EBST and hanging wire test were performed to evaluate the neurobehavioral function. The cresyl violet staining was used to detect cerebral infarction. No statistical significance in body weight of the five groups was found (Fig. 5B). Compared to IS and IS-MP groups, injection of RIL4 and MP/RIL4 significantly decreased the neurological deficits of stroke mice (Fig. 5C–E). Mice treated with MP/RIL4 showed reduced brain atrophy volume compared with other groups (Fig. 5F and G). These results supported that the “immunomodulator” had neurorestorative effects on promoting neurobehavioral function and reducing brain atrophy volume.

### 3.5 Immunoregulation effect of the “immunomodulator” *in vivo*

**Fig. 6:**
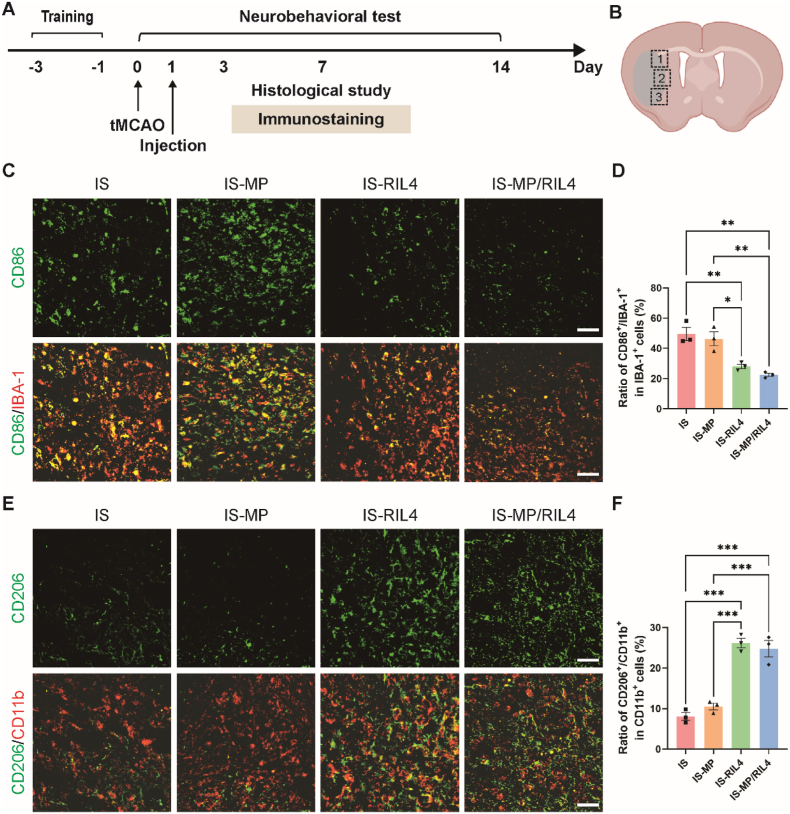
**Immunoregulation effect of the “immunomodulator” at day 7 post-tMCAO *in vivo*.** (A) Experimental diagram. (B) Diagram of the analyzed peri-infarct regions of 1, 2 and 3. The pattern was created with BioRender.com. (C, D) Representative co-immunostaining images of CD86 and IBA-1 and quantitative analysis of the ratio of CD86^+^/IBA-1^+^ cells in IBA-1^+^ cells. Scale bar = 50 μm. n = 3 mice. (E, F) Representative co-immunostaining images of CD206 and CD11b and quantitative analysis of the ratio of CD206^+^/CD11b^+^ cells in CD11b^+^ cells. Scale bar = 50 μm. n = 3 mice. All data are presented as mean ± SEM, ∗*p* < 0.05, ∗∗*p* < 0.01, ∗∗∗*p* < 0.001.

**Fig. 7:**
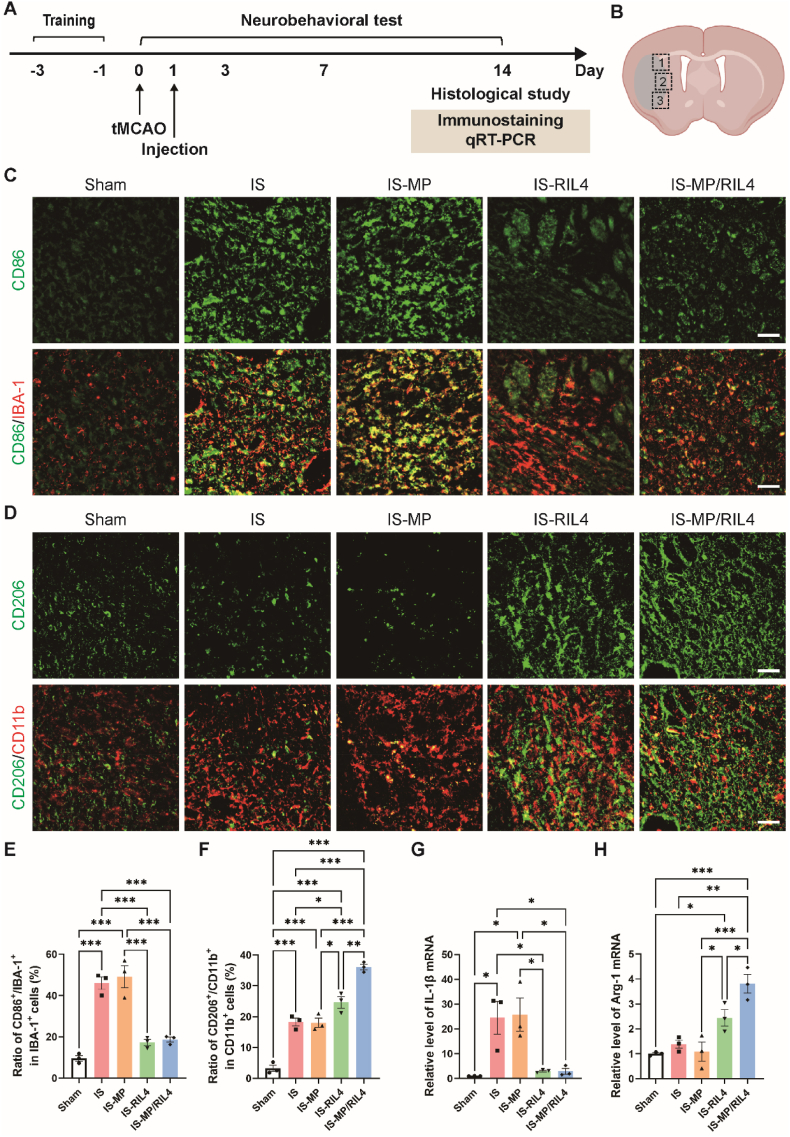
**Immunoregulation effect of the “immunomodulator” at day 14 post-tMCAO *in vivo*.** (A) Experimental diagram. (B) Diagram of the analyzed peri-infarct regions of 1, 2 and 3. The pattern was created with BioRender.com. (C, E) Representative co-immunostaining images of CD86 and IBA-1 and quantitative analysis of the ratio of CD86^+^/IBA-1^+^ cells in IBA-1^+^ cells. Scale bar = 50 μm. n = 3 mice. (D, F) Representative co-immunostaining images of CD206 and CD11b and quantitative analysis of the ratio of CD206^+^/CD11b^+^ cells in CD11b^+^ cells. Scale bar = 50 μm. n = 3 mice. RT-PCR analysis of (G) pro-inflammatory marker (IL-1β) and (H) anti-inflammatory marker (Arg-1) expression of MP, RIL4 and MP/RIL4 injection after ischemic stroke. n = 3 mice. All data are presented as mean ± SEM, ∗*p* < 0.05, ∗∗*p* < 0.01, ∗∗∗*p* < 0.001.

Regulating the activation and polarization of microglia, the resident immune cells within the brain are critical for attenuating subsequent inflammatory response and promoting tissue repair following ischemic stroke [23]. Microglia primarily polarize into pro-inflammatory phenotype, secreting high pro-inflammatory mediators like TNF-α, IL-1β, and iNOS, promoting cytotoxic oxidative stress and neuronal death. Conversely, microglia can also polarize into anti-inflammation type, which could release anti-inflammatory molecules including IL-10, Arg-1, and TGF-β, contributing to debris clearance, suppression of inflammation, and neuro-regeneration, thus contributing to function recovery. To test the immunoregulation effect of our “immunomodulator”, mice were sacrificed at day 7 and day 14 post-tMCAO to observe the neuroinflammatory level. (Fig. 6, Fig. 7A-B). CD86 is a marker of pro-inflammation, which was co-expressed with IBA-1^+^ microglia and increased after tMCAO, and reversed by RIL4 and MP/RIL4 treatment (Fig. 6C and D). Conversely, CD206, an anti-inflammatory marker, was co-expressed with CD11b^+^ microglia and increased in RIL4 and MP/RIL4 groups compared to IS and IS-MP groups (Fig. 6E and F). Similar results were also obtained at day 14 (Fig. 7C–F). Moreover, the MP/RIL4 group exhibited increased anti-inflammatory factors of CD206 than RIL4 group at day 14 post-tMCAO (Fig. 7F), and the increased CD206 only existed in ipsilateral hemisphere (Supp. Fig. 3A). The results of RT-PCR analysis verified the anti-inflammatory effect of MP/RIL4 at day 14 (Fig. 7G and H), which also matched the results of BV2 cells *in vitro*. Improved immune microenvironment may contribute to reducing cell death. Thus, we continued to detect the density of neurons after tMCAO at day 7 and day 14 (Supp. Fig. 3B). Results showed that IS-RIL4 and IS-MP/RIL4 groups increased the number of Neun^+^ neurons at both 7 and 14 days after tMCAO, and increased in a time-dependent manner (Supp. Figs. 3C–E). The anti-inflammatory effect of MP/RIL4 was also in a time-dependent manner through re-analysis of day 7 and day 14 data (Supp. Figs. 3F–G). The study suggested that MP/RIL4 had a significant anti-inflammatory effect and potentially improved microglial function, making it a promising approach for reducing brain pathologies involving neuroinflammation.

As a classical cytokine for modulating microglial polarization, IL-4 has a very short half-life period, and its effective time in the body is only several minutes [58]. Continuous administration of lateral ventricle injection or nasal administration is often required for more than 7 d, which complicates the administration procedure [23,25]. It is worth noting that we only injected once at day 1 after tMCAO, and the MP/RIL4 held a better anti-inflammatory ability than naked RIL4, which manifested the long-term role of microspheres for targeted sustained release of IL-4.

### 3.6 The “immunomodulator” improved angiogenesis and neurogenesis *in vitro* and *in vivo*

In the chronic stage of stroke, it will initiate a repair mechanism involving angiogenesis and neurogenesis, both of which play critical roles in promoting functional recovery post-stroke [59]. Angiogenesis refers to the formation of new blood vessel structures based on the existing vasculature, improving blood supply to the ischemic region, delivering essential oxygen and nutrients, removing metabolic waste, and helping to reduce inflammation and promote tissue repair after injury [60]. Neurogenesis, on the other hand, involves the ability of neural stem cells or progenitor cells in subventricular zone (SVZ), to differentiate into new neurons and supporting cells (such as astrocytes and oligodendrocytes) [[61], [62], [63]]. Previous studies showed that angiogenesis generally started at 3 days after stroke, and lasted for several weeks [[64], [65], [66]], and neurogenesis began at the first week after stroke and lasted for weeks to months [[67], [68], [69]]. Thus, limited angiogenesis and neurogenesis happens before 7 days after stroke. So, we tested angiogenesis and neurogenesis at 14 days after stroke. However, the angiogenesis and neurogenesis after stroke are limited due to the lack of effective linkages between immune cells and neurovascular components.

**Fig. 8:**
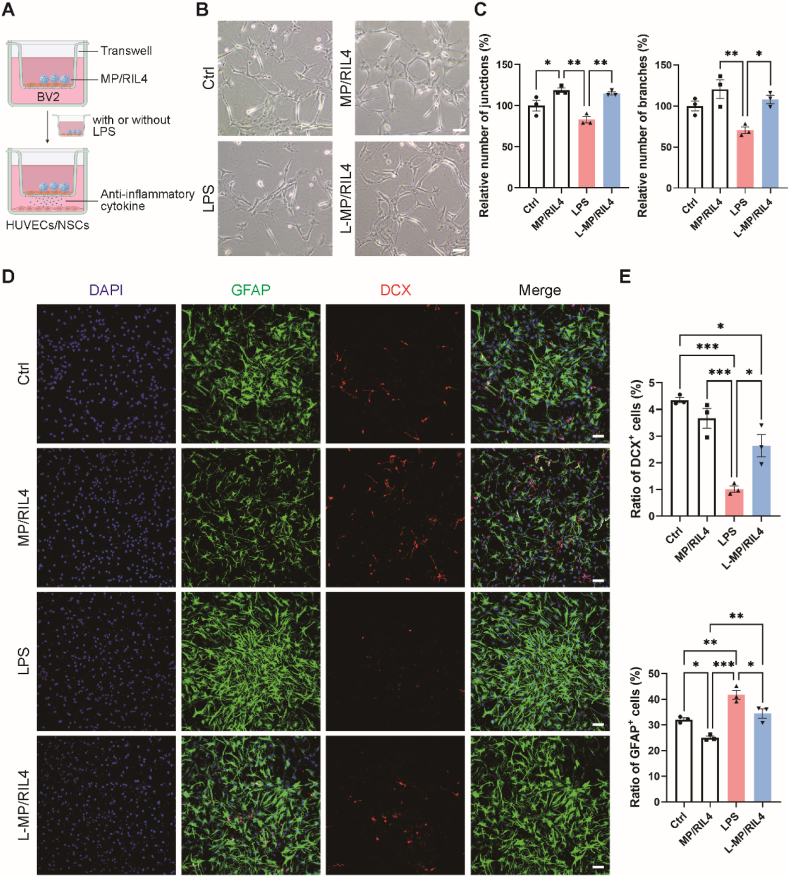
**The “immunomodulator” regulated immune-neurovascular crosstalk *in vitro*.** (A) Experimental diagram of BV2-HUVECs/NSCs cell interaction model. The schematic diagram was created with BioRender.com. (B–C) Representative images and analysis of tube formation of HUVECs which were co-cultured with BV2 microglia or MP/RIL4-treated BV2 microglia with or without LPS. Scale bar = 50 μm. n = 3. (D–E) Representative immunostaining images and differentiation of NSCs which were co-cultured with BV2 microglia or MP/RIL4-treated BV2 microglia with or without LPS. Scale bar = 50 μm. n = 3. All data are presented as mean ± SEM, ∗*p* < 0.05, ∗∗*p* < 0.01, ∗∗∗*p* < 0.001.

**Fig. 9:**
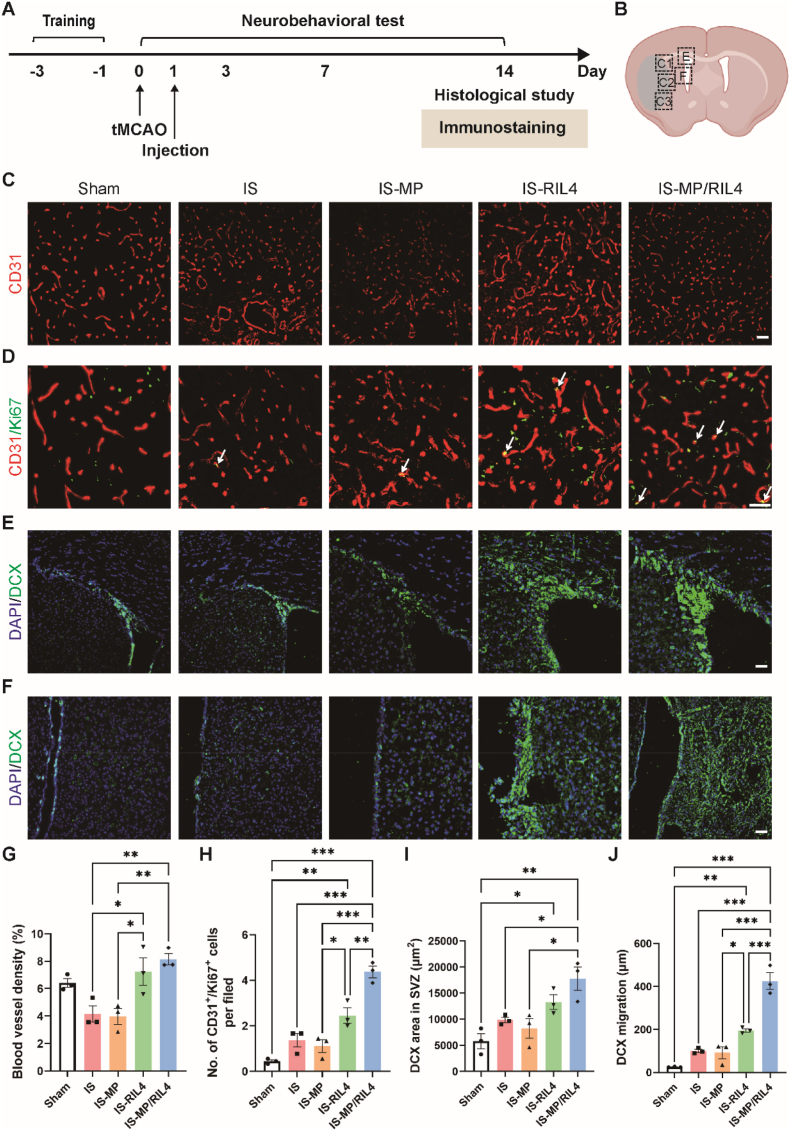
**The “immunomodulator” improved angiogenesis and neurogenesis of ischemic stroke mice.** (A) Experimental diagram. (B) Diagram of the analyzed peri-infarct regions of C1, C2, C3, E and F in corresponding brain slices. The pattern was created with BioRender.com. Representative images of (C) CD31^+^ blood vessels and (D) CD31^+^/Ki67^+^ vessels. Scale bar = 50 μm. n = 3 mice. Representative images of DCX^+^ cells in (E) SVZ and (F) striatum. Scale bar = 50 μm. n = 3 mice. Quantitative analysis of (G) blood vessel density and (H) numbers of CD31^+^/Ki67^+^ vessels, (I) DCX area in SVZ and (J) DCX migration. n = 3 mice. All data are presented as mean ± SEM, ∗*p* < 0.05, ∗∗*p* < 0.01, ∗∗∗*p* < 0.001.

The immunomodulator we produced can also provide structural support in infarct area and it may play a role in bridging the polarized microglia and neurovascular unit. To investigate the immune-neurovascular crosstalk regulated by MP/RIL4 microspheres *in vitro*, we employed a transwell co-culture system (Fig. 8A). BV2 microglia were seeded on transwell inserts and incubated with MP/RIL4 for 1 h. After that, BV2 cells were treated with or without LPS, and then co-cultured with HUVECs or NSCs for 24 h. We found that MP/RIL4 alone enhanced the number of tube junctions. LPS treatment reduced the number of tube junctions and branches compared to the control group, but L-MP/RIL4 rescued the impaired tube formation (Fig. 8B and C). Additionally, L-MP/RIL4 increased the number of DCX^+^ neuroblasts and decreased the number of GFAP^+^ astrocytes compared to LPS group (Fig. 8D and E). The results demonstrated that MP/RlL4 treated BV2 microglia increased tube formation of HUVECs, as well as enhanced NSCs differentiation to neuroblast, which provided the direct evidence of immune-neurovascular crosstalk regulated by MP/RlL4 microspheres. By performing double immunostaining of CD31 and IBA-1 *in vivo*, as well as DCX and CD11b, we confirmed that both neuroblast in the SVZ and endothelial cells in the striatum physically contact with immune cells (microglia in this study) (Supp. Figs. 3H-I), suggesting the potential interaction between immune cells and endothelial cells, as well as immune cells and neurons. We then continued to detect the angiogenesis and neurogenesis of ischemic mice by immunostaining (Fig. 9A and B). As shown in Fig. 9C–D, and G-H, RIL4 and MP/RIL4 treated mice had increased density of CD31^+^ blood vessels in periinfarction area compared to control mice. The number of CD31^+^/Ki67^+^ endothelial cells raised in MP/RIL4 treated mice, suggests the effective angiogenesis ability of RIL4-loaded microspheres. Additionally, we observed an augmented presence of DCX^+^ neuroblasts in the SVZ of ischemic mice following MP/RIL4 treatment, but not in other groups (Fig. 9E–I). Moreover, the MP/RIL4 group showed better migration potential to injured areas than other groups (Fig. 9F–J). The injection of MP/RIL4 increased angiogenesis and neurogenesis in ischemic stroke mice.

Angiogenesis and neurogenesis work collaboratively to restore the integrity of neurovascular unit, and support neuroprotection and tissue repair [70]. IL4-polarized microglia could ameliorate ischemic injury by promoting angiogenesis via secretion of miRNA-26 accumulated exosomes [71]. Through transwell or conditioned medium of IL4-treated microglia to deal with adult neural stem/progenitor cells (NSPCs), Jiang et al. found that it could promote NSPCs proliferation, survival, and differentiation into neurons and oligodendrocytes via PI3K-Akt pathway [72]. Besides, Zhang et al. identified that IL4-driven microglia in the hippocampus characterized by high expression of Arg1, triggered BDNF-dependent neurogenesis in a mouse model of chronic mild stress [73]. In addition to the role of RIL4 for angiogenesis and neurogenesis, PVA microspheres were also provided as scaffolds in infarct areas, which may be the reason why MP/RIL4 group showed a more superior capability to promote regeneration than RIL4 group.

## 4 Conclusion

In this study, we developed an immune-neurovascular crosstalk bridge via the immunomodulatory microsphere (MP/RIL4), which could induce the transformation of microglia into an anti-inflammatory phenotype, and link their interaction with neurovascular unit, ultimately succeeding to promote angiogenesis and neurogenesis. The immunomodulator not only downregulated the expression of pro-inflammatory markers (IL-1β, iNOS, CD86) and upregulated anti-inflammatory markers (IL-10, Arg-1, CD206) both in *in vitro* cellular model and *in vivo* stroke model to improve the local immune milieu, but it also reduced brain atrophy volume in stroke mice, enhanced neurological behavioral functions, and precisely coordinated immune cells with the neurovascular unit to facilitate both angiogenesis and neurogenesis. In summary, this targeted microglia-loading immune modulator system offers a novel approach for enhancing the interaction between the immune system and neurovascular unit, thereby boosting neurovascular regeneration.

## CRediT authorship contribution statement

**Tongtong Xu:** Writing – original draft, Data curation. **Lin Gan:** Investigation. **Wei Chen:** Methodology. **Dandan Zheng:** Methodology. **Hanlai Li:** Methodology. **Shiyu Deng:** Methodology. **Dongliang Qian:** Software. **Tingting Gu:** Methodology. **Qianyuan Lian:** Software. **Gracie Shen:** Data curation. **Qingzhu An:** Writing – review & editing. **Wanlu Li:** Writing – review & editing. **Zhijun Zhang:** Writing – review & editing. **Guo-Yuan Yang:** Writing – review & editing. **Huitong Ruan:** Writing – review & editing, Writing – original draft, Supervision, Conceptualization. **Wenguo Cui:** Writing – review & editing, Supervision, Conceptualization. **Yaohui Tang:** Writing – review & editing, Supervision, Conceptualization.

## Ethics approval and consent to participate

Animal experiments followed guidelines of the Institutional Animal Care and Use Committee (IACUC) of Shanghai Jiao Tong University, Shanghai, China (No. 2023011).

## Declaration of competing interest

The authors declare that they have no known competing financial interests or personal relationships that could have appeared to influence the work reported in this paper.

## References

[1] Xiong Y. (2022). The role of the immune microenvironment in bone, cartilage, and soft tissue regeneration: from mechanism to therapeutic opportunity. Mil Med Res.

[2] Xiang L. (2023). Motion lubrication suppressed mechanical activation via hydrated fibrous gene patch for tendon healing. Sci. Adv..

[3] Baldino L. (2016). Regeneration techniques for bone-to-tendon and muscle-to-tendon interfaces reconstruction. Br. Med. Bull..

[4] Quint J.P. (2021). In vivo printing of nanoenabled scaffolds for the treatment of skeletal muscle injuries. Adv. Healthcare Mater..

[5] Huang Y. (2020). Crosstalk between inflammation and the BBB in stroke. Curr. Neuropharmacol..

[6] Farfara D. (2019). Knockdown of circulating C1 inhibitor induces neurovascular impairment, glial cell activation, neuroinflammation, and behavioral deficits. Glia.

[7] Li W. (2024). T cell senescence may contribute to immunothrombosis via Th17 immune transition in COVID-19. Sci. Bull..

[8] Wang C. (2021). Selective removal of astrocytic APOE4 strongly protects against tau-mediated neurodegeneration and decreases synaptic phagocytosis by microglia. Neuron.

[9] Planas A.M. (2024). Role of microglia in stroke. Glia.

[10] Hughes A.N., Appel B. (2020). Microglia phagocytose myelin sheaths to modify developmental myelination. Nat. Neurosci..

[11] Xu T. (2022). The roles of microglia and astrocytes in myelin phagocytosis in the central nervous system. J. Cerebr. Blood Flow Metabol..

[12] Shen H. (2023). Microglia and astrocytes mediate synapse engulfment in a MER tyrosine kinase-dependent manner after traumatic brain injury. Neural Regen. Res..

[13] Li C. (2023). Regulated macrophage immune microenvironment in 3D printed scaffolds for bone tumor postoperative treatment. Bioact. Mater..

[14] Zheng D. (2023). Advances in extracellular vesicle functionalization strategies for tissue regeneration. Bioact. Mater..

[15] Podlasek A. (2022). To bridge or not to bridge: summary of the new evidence in endovascular stroke treatment. Stroke Vasc. Neurol..

[16] Shen Y. (2022). Current treatments after spinal cord injury: cell engineering, tissue engineering, and combined therapies. Smart Med..

[17] Liu H. (2023). Stem cell niches functionalized strategies for organ regeneration and manufacturing. Innovat. Med..

[18] Araki T., Ikegaya Y., Koyama R. (2021). The effects of microglia- and astrocyte-derived factors on neurogenesis in health and disease. Eur. J. Neurosci..

[19] Huang W. (2023). Microglia-mediated neurovascular unit dysfunction in Alzheimer's Disease. J. Alzheimers Dis..

[20] Zeng J. (2022). The mechanism of microglia-mediated immune inflammation in ischemic stroke and the role of natural botanical components in regulating microglia: a review. Front. Immunol..

[21] Ma Y. (2017). The biphasic function of microglia in ischemic stroke. Prog. Neurobiol..

[22] Colonna M., Butovsky O. (2017). Microglia function in the central nervous system during health and neurodegeneration. Annu. Rev. Immunol..

[23] Liu X. (2016). Interleukin-4 is essential for microglia/macrophage M2 polarization and long-term recovery after cerebral ischemia. Stroke.

[24] He Y. (2020). IL-4 switches microglia/macrophage M1/M2 polarization and alleviates neurological damage by modulating the JAK1/STAT6 pathway following ICH. Neuroscience.

[25] Xu J. (2020). IL-4/STAT6 signaling facilitates innate hematoma resolution and neurological recovery after hemorrhagic stroke in mice. Proc. Natl. Acad. Sci. U. S. A..

[26] Chen W. (2023). A matrix-metalloproteinase-responsive hydrogel system for modulating the immune microenvironment in myocardial infarction. Adv. Mater..

[27] Sun X. (2023). Targeted regulation of neuroinflammation via nanobiosignaler for repairing the central nerve system injuries. Nano Res..

[28] Zheng D. (2024). Hydrogen ion capturing hydrogel microspheres for reversing inflammaging. Adv. Mater..

[29] Deng S. (2023). Roles of ependymal cells in the physiology and pathology of the central nervous system. Aging Dis..

[30] Xu T. (2022). Introduction to biomedical engineering in stroke diagnosis and treatment. Stroke.

[31] Choi S.H., Park T.G. (2006). G-CSF loaded biodegradable PLGA nanoparticles prepared by a single oil-in-water emulsion method. Int. J. Pharm..

[32] Chen W. (2023). Circadian clock regulation via biomaterials for nucleus pulposus. Adv. Mater..

[33] Ruan H. (2019). A dual-bioresponsive drug-delivery depot for combination of epigenetic modulation and immune checkpoint blockade. Adv. Mater..

[34] Shi X. (2021). Stroke subtype-dependent synapse elimination by reactive gliosis in mice. Nat. Commun..

[35] Li Y. (2022). M2 microglia-derived extracellular vesicles promote white matter repair and functional recovery via miR-23a-5p after cerebral ischemia in mice. Theranostics.

[36] Suo Q. (2023). Optogenetic activation of astrocytes reduces blood-brain barrier disruption via IL-10 in stroke. Aging Dis..

[37] Wang C. (2022). Targeted delivery of fat extract by platelet membrane-cloaked nanocarriers for the treatment of ischemic stroke. J. Nanobiotechnol..

[38] Tian M., Ma Z., Yang G.Z. (2024). Micro/nanosystems for controllable drug delivery to the brain. The Innovation.

[39] Chen L. (2024). Mitochondrial-oriented injectable hydrogel microspheres maintain homeostasis of chondrocyte metabolism to promote subcellular therapy in osteoarthritis. Research.

[40] Li X. (2023). In situ sustained macrophage-targeted nanomicelle-hydrogel microspheres for inhibiting osteoarthritis. Research.

[41] Lu H., Wang Y., Yu R. (2023). Immune cell membrane-coated nanoparticles for targeted myocardial ischemia/reperfusion injury therapy. Innovat. Med..

[42] Ransohoff R.M. (2016). A polarizing question: do M1 and M2 microglia exist?. Nat. Neurosci..

[43] Ma H. (2023). Microglia exhibit distinct heterogeneity rather than M1/M2 polarization within the early stage of acute ischemic stroke. Aging Dis..

[44] Chen X. (2020). Deficiency of anti-inflammatory cytokine IL-4 leads to neural hyperexcitability and aggravates cerebral ischemia-reperfusion injury. Acta Pharm. Sin. B.

[45] Gadani S.P. (2012). IL-4 in the brain: a cytokine to remember. J. Immunol..

[46] Ryu K.Y. (2019). Dasatinib regulates LPS-induced microglial and astrocytic neuroinflammatory responses by inhibiting AKT/STAT3 signaling. J. Neuroinflammation.

[47] Bi W. (2023). PSMC5 regulates microglial polarization and activation in LPS-induced cognitive deficits and motor impairments by interacting with TLR4. J. Neuroinflammation.

[48] Cai L. (2022). ACT001 attenuates microglia-mediated neuroinflammation after traumatic brain injury via inhibiting AKT/NFκB/NLRP3 pathway. Cell Commun. Signal..

[49] Ruan H. (2018). A novel peptide ligand RAP12 of LRP1 for glioma targeted drug delivery. J. Contr. Release.

[50] Brifault C. (2019). LRP1 deficiency in microglia blocks neuro-inflammation in the spinal dorsal horn and neuropathic pain processing. Glia.

[51] Nguyen V.H., Lee B.J. (2017). Protein corona: a new approach for nanomedicine design. Int. J. Nanomed..

[52] Jearanaiwitayakul T. (2020). Nanodelivery system enhances the immunogenicity of dengue-2 nonstructural protein 1, DENV-2 NS1. Vaccine.

[53] Tang Y. (2022). The effect of drug loading and multiple administration on the protein corona formation and brain delivery property of PEG-PLA nanoparticles. Acta Pharm. Sin. B.

[54] Engelter S.T. (2008). The clinical significance of diffusion-weighted MR imaging in stroke and TIA patients. Swiss Med. Wkly..

[55] Ruan H. (2023). Engineered extracellular vesicles for ischemic stroke treatment. The Innovation.

[56] Li P. (2022). Altered excitability of motor neuron pathways after stroke: more than upper motor neuron impairments. Stroke Vasc. Neurol..

[57] Tian D.S. (2023). Prevalence and risk factors of stroke in China: a national serial cross-sectional study from 2003 to 2018. Stroke Vasc. Neurol..

[58] Liu H. (2019). Efficacy of pulmonary transplantation of engineered macrophages secreting IL-4 on acute lung injury in C57BL/6J mice. Cell Death Dis..

[59] Li L. (2021). Astragaloside IV promotes microglia/macrophages M2 polarization and enhances neurogenesis and angiogenesis through PPARγ pathway after cerebral ischemia/reperfusion injury in rats. Int. Immunopharm..

[60] Yu W. (2021). Connexin43 promotes angiogenesis through activating the HIF-1α/VEGF signaling pathway under chronic cerebral hypoperfusion. J. Cerebr. Blood Flow Metabol..

[61] Koh S.H., Park H.H. (2017). Neurogenesis in stroke recovery. Transl. Stroke Res..

[62] Jiang C. (2016). Progesterone changes VEGF and BDNF expression and promotes neurogenesis after ischemic stroke. Mol. Neurobiol..

[63] Xiong X.Y., Liu L., Yang Q.W. (2016). Functions and mechanisms of microglia/macrophages in neuroinflammation and neurogenesis after stroke. Prog. Neurobiol..

[64] Pradillo J.M. (2021). Influence of metabolic syndrome on post-stroke outcome, angiogenesis and vascular function in old rats determined by dynamic contrast enhanced MRI. J. Cerebr. Blood Flow Metabol..

[65] Hu G.J. (2017). Effect of combined VEGF(165)/SDF-1 gene therapy on vascular remodeling and blood perfusion in cerebral ischemia. J. Neurosurg..

[66] Xie W. (2023). Neutrophil-derived cathelicidin promotes cerebral angiogenesis after ischemic stroke. J. Cerebr. Blood Flow Metabol..

[67] Var S.R. (2021). Microglia and macrophages in neuroprotection, neurogenesis, and emerging therapies for stroke. Cells.

[68] Taupin P. (2006). Stroke-induced neurogenesis: physiopathology and mechanisms. Curr. Neurovascular Res..

[69] Colitti N. (2022). Long-term intranasal nerve growth factor treatment favors neuron formation in de novo brain tissue. Front. Cell. Neurosci..

[70] Li G. (2020). Construction of dual-biofunctionalized chitosan/collagen scaffolds for simultaneous neovascularization and nerve regeneration. Research.

[71] Tian Y. (2019). IL-4-polarized BV2 microglia cells promote angiogenesis by secreting exosomes. Adv. Clin. Exp. Med..

[72] Jiang X. (2022). The secretome of microglia induced by IL-4 of IFN-γ differently regulate proliferation, differentiation and survival of adult neural stem/progenitor cell by targeting the PI3K-Akt pathway. Cytotechnology.

[73] Zhang J. (2021). IL4-driven microglia modulate stress resilience through BDNF-dependent neurogenesis. Sci. Adv..

