# Supplementary material for: Bridging immune-neurovascular crosstalk via the immunomodulatory microspheres for promoting neural repair
Source: Bioact Mater. 2024 Nov 8;44:558–71. doi: 10.1016/j.bioactmat.2024.10.031 (PMC11583666; doi:10.1016/j.bioactmat.2024.10.031)
Supplement: Multimedia component 1 [file mmc1.docx]

**Bridging immune-neurovascular crosstalk via the immunomodulatory microspheres for promoting neural repair**

**Supplementary Table 1.** Sample size of *in vivo* study

|  | Total number | Neurobehavioral test | Number of mice sacrificed at day 7 |
| --- | --- | --- | --- |
| Sham | 10 | 10 (Immunostaining: n=3; RT-PCR: n=3) | N.A. |
| IS | 10 | 7 (Immunostaining: n=3; RT-PCR: n=3) | 3 |
| IS-MP | 11 | 8 (Immunostaining: n=3; RT-PCR: n=3) | 3 |
| IS-RIL4 | 12 | 9 (Immunostaining: n=3; RT-PCR: n=3) | 3 |
| IS-MP/RIL4 | 10 | 7 (Immunostaining: n=3; RT-PCR: n=3) | 3 |

**Supplementary Figure 1**

**
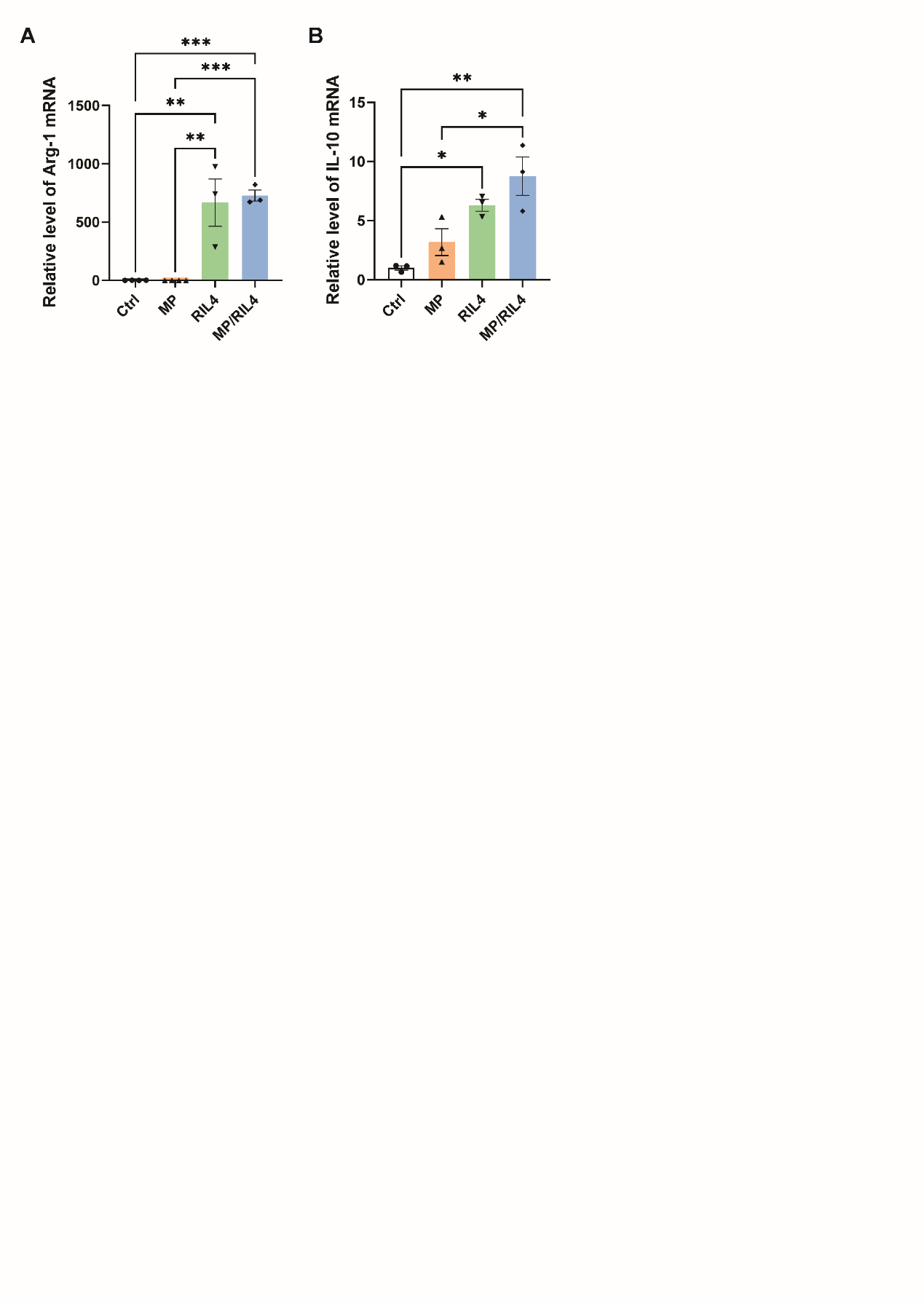
**

**Supplementary Figure 1.** Quantitative RT-PCR analysis of (A) Arg-1, (B) IL-10 expression of PBS, MP, RIL4 and MP/RIL4 co-cultured with BV2 microglia after 24 h. n=3-4. All data are presented as mean ± SEM, **p* < 0.05, ***p* < 0.01, ****p* < 0.001.

**Supplementary Figure 2**

**
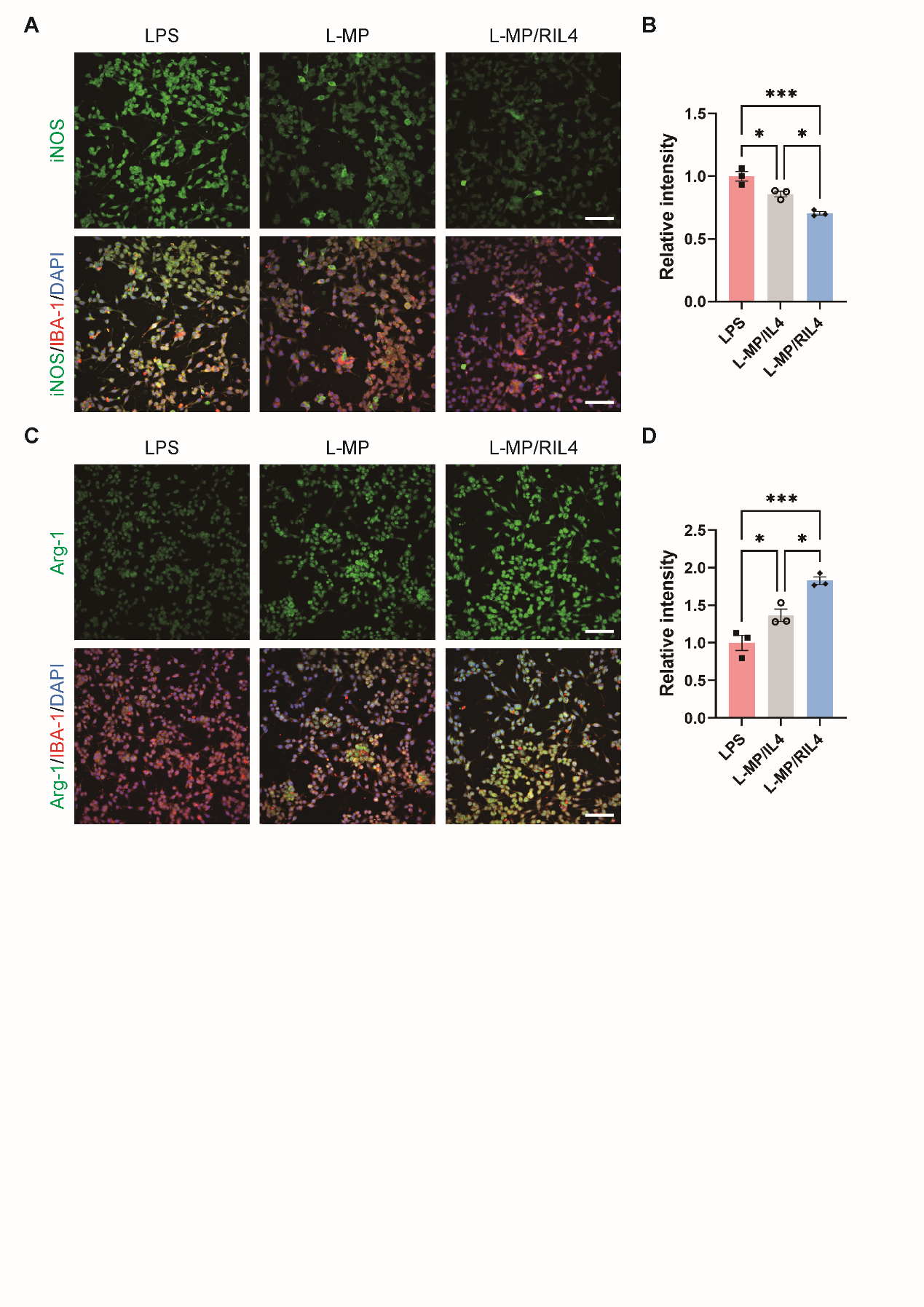
**

**Supplementary Figure 2.** (A, B) Representative immunostaining images and relative total fluorescence intensity of iNOS with IBA-1 after PBS, MP, MP/IL4, RIL4 and MP/RIL4 co-cultured with LPS stimulated BV2 microglia. Scale bar=100 μm. n=3. (C, D) Representative images and quantification of Arg-1 with IBA-1 after PBS, MP, MP/IL4, RIL4 and MP/RIL4 co-cultured with LPS stimulated BV2 microglia. Scale bar=100 μm. n=3. All data are presented as mean ± SEM, *p < 0.05, ***p < 0.001.

**Supplementary Figure 3**

**
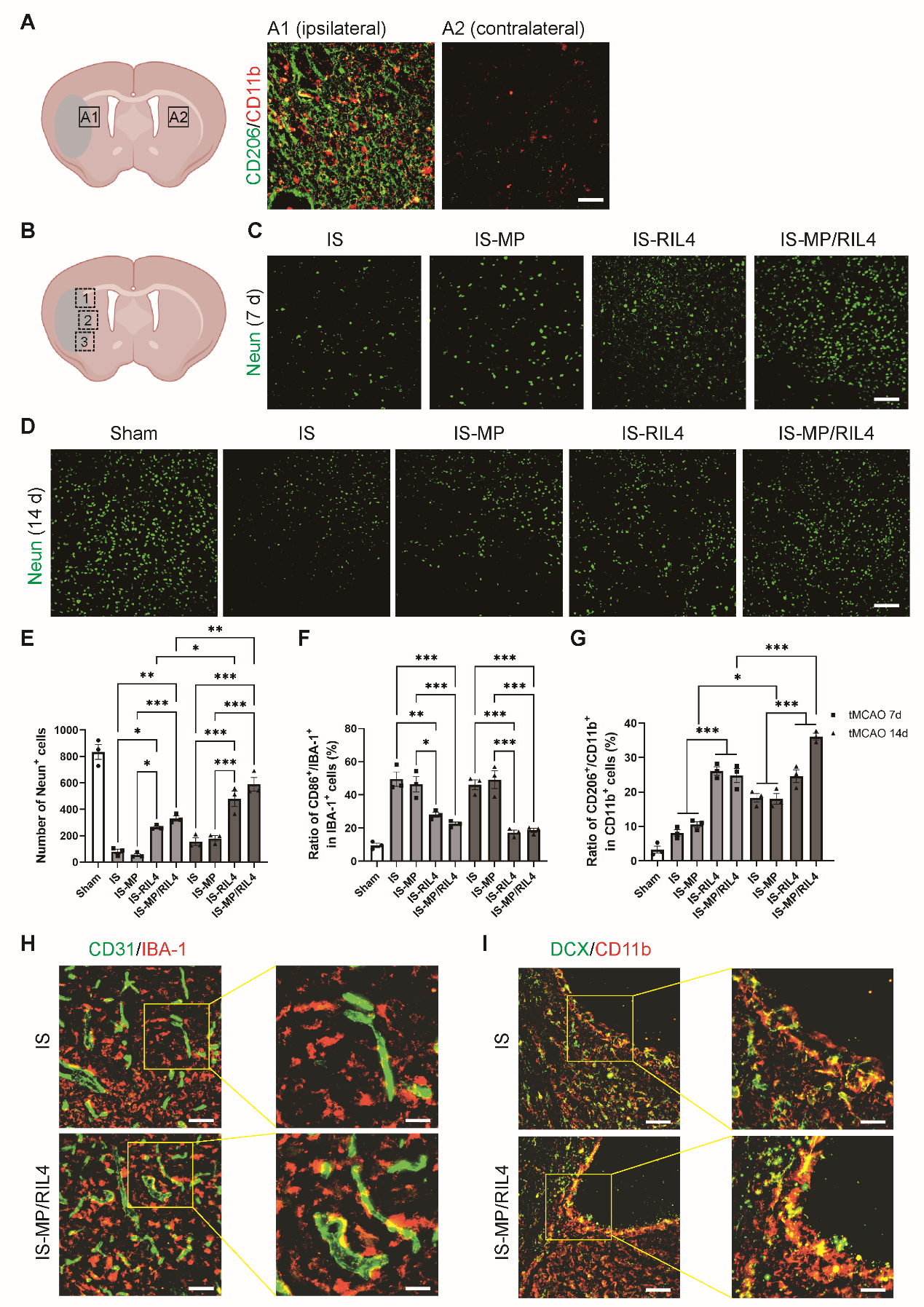
**

**Supplementary Figure 3.** (A) Representative images of CD206 co-stained with CD11b of ipsilateral (A1) and contralateral (A2) hemisphere at day 14 post-tMCAO. Scale bar=50 μm. (B) Diagram of the analyzed peri-infarct three regions of Neun+ cells. Representative staining images of Neun in day 7 (C) and day 14 (D) after tMCAO. Scale bar=100 μm. Combined statistical analysis of the number of Neun+ cells (E), ratio of CD86^+^/IBA-1^+^ cells in IBA-1^+^ cells (F), and ratio of CD206^+^/CD11b^+^ cells in CD11b^+^ cells (G) at day 7 and day 14 after tMCAO. n=3 mice. Immunostaining showed the crosstalk of CD31+ endothelial cells with IBA-1+ microglia (H), and DCX+ neuroblast with CD11b+ microglia (I) at day 14 post-tMCAO. Scale bar=50 μm (left), 10 μm (right). All data are presented as mean ± SEM, **p* < 0.05, ***p* < 0.01, ****p* < 0.001.

**Supplementary Figure 4**

**
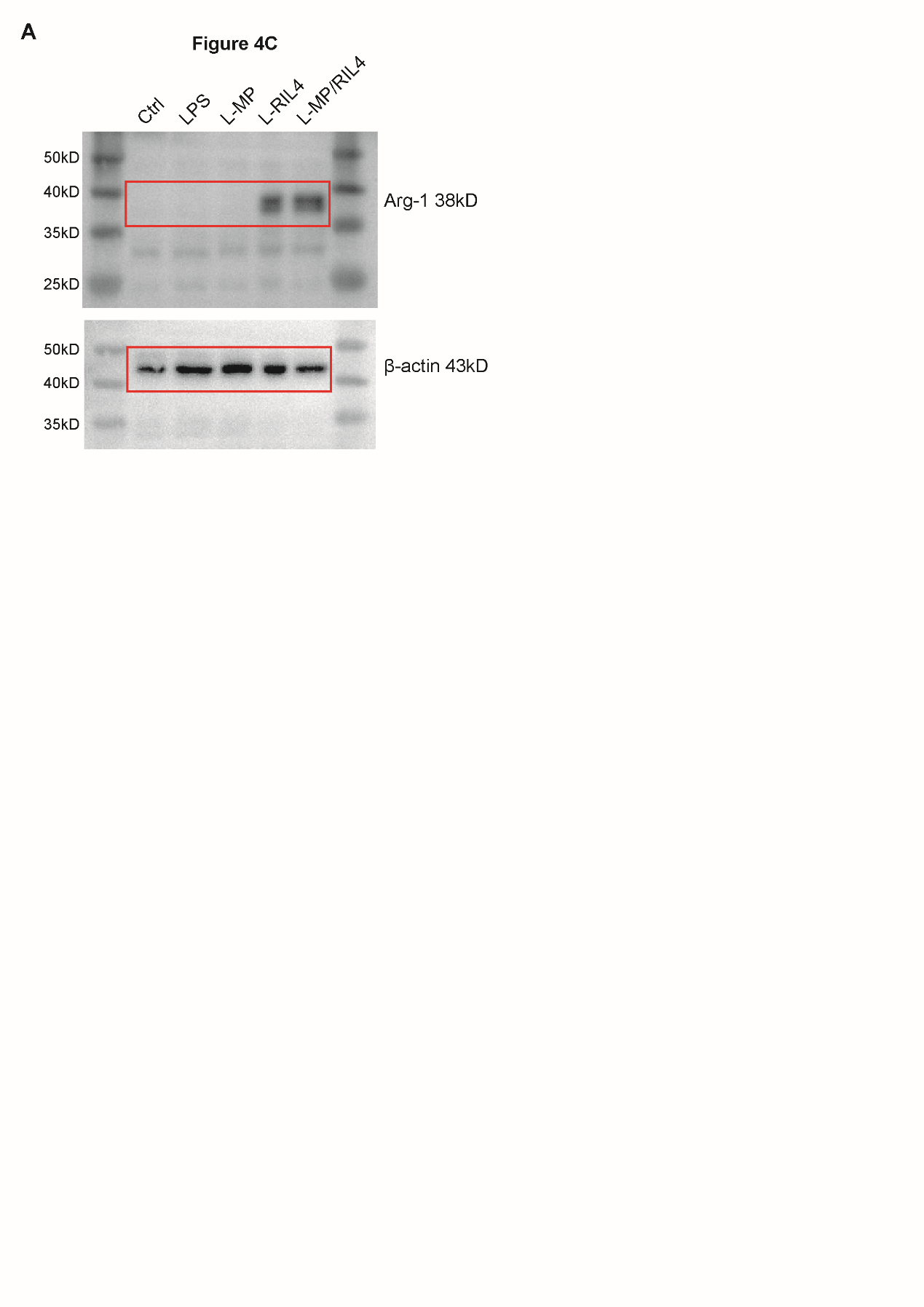
**

**Supplementary Figure 4. WB raw data.**
